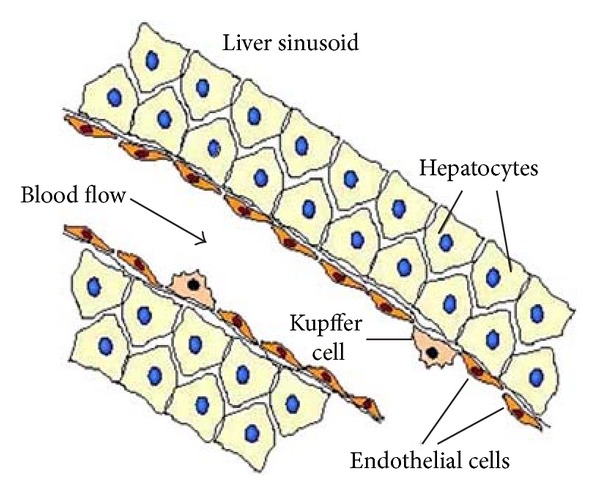# Analytical Solutions for the Mathematical Model Describing the Formation of Liver Zones via Adomian's Method

**DOI:** 10.1155/2013/547954

**Published:** 2013-08-28

**Authors:** Abdelhalim Ebaid

**Affiliations:** Department of Mathematics, Faculty of Science, University of Tabuk, P.O. Box 741, Tabuk 71491, Saudi Arabia

## Abstract

The formation of liver zones is modeled by a system of two integropartial differential equations. In this research, we introduce the mathematical formulation of these integro-partial differential equations obtained by Bass et al. in 1987. For better understanding of this mathematical formulation, we present a medical introduction for the liver in order to make the formulation as clear as possible. In applied mathematics, the Adomian decomposition method is an effective procedure to obtain analytic and approximate solutions for different types of operator equations. This Adomian decomposition method is used in this work to solve the proposed model analytically. The stationary solutions (as time tends to infinity) are also obtained through it, which are in full agreement with those obtained by Bass et al. in 1987.

## 1. Introduction

**Figure 1:**
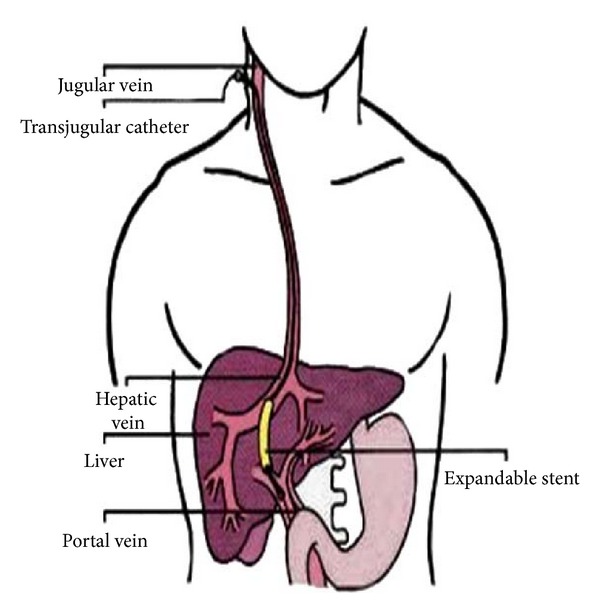


**Figure 2:**
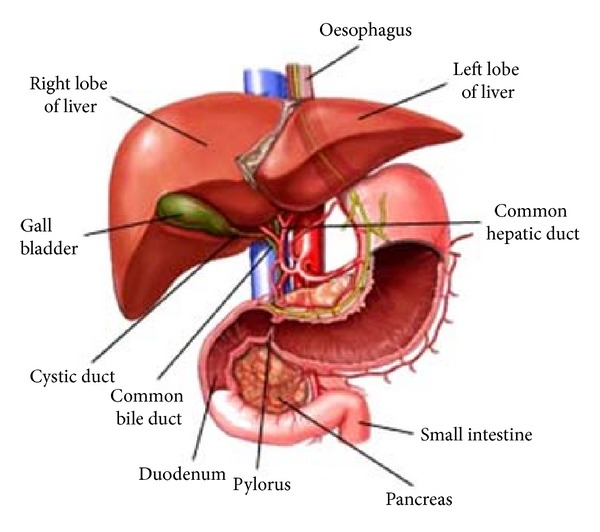


The dark-red liver is the body's largest single gland (1 to 1.5 kg). It is a metabolic middleman because it takes up and secretes more than 500 different kinds of molecules. The liver stores and releases glucose, keeping blood glucose levels relatively constant. The location of the liver reflects its middleman's role (Figure 1). The gland lies between the diaphragm above and the stomach and intestines below (Figure 2). Glucose and many other molecules enter the liver through the hepatic portal vein, and their products go directly through the inferior vena cava to the heart and lungs and then into the systemic circulation. The liver takes its dome-like shape from the diaphragm, which covers its superior surface, called diaphragmatic surface. The sagittal fossa divides the liver into two great lobes, right and left. The right lobe is larger and displays two smaller quadrate and caudate lobes on its visceral surface, defined by gallbladder and inferior vena cava, respectively [1]. The hepatic veins drain into the inferior vena cava arising from the posterior part of diaphragmatic surface of the liver. Visceral peritoneum binds the liver to the diaphragm and to the posterior wall of the abdomen.

**Figure 3:**
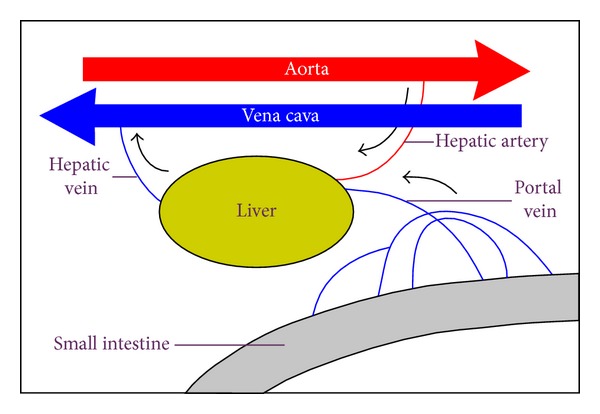


**Figure 4:**
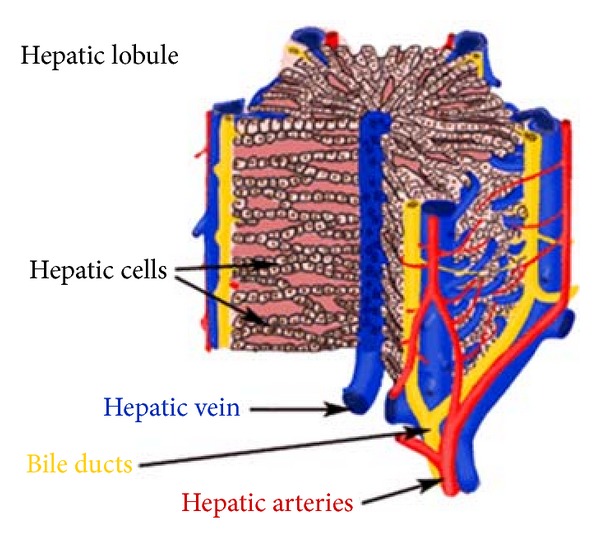


Although there is an extensive bare area on the diaphragmatic surface of the liver where the peritoneum does not reach, the connective tissue attaches this area directly to the diaphragm. Most of the blood to the liver (70–80%) comes from the portal vein, and the smaller percentage is supplied by the hepatic artery (Figure 3). All the materials absorbed via the intestines reach the liver through the portal vein, except the complex lipids which are transported mainly by lymph vessels. The position of the liver in the circulatory system is optimal for gathering, transforming, and accumulating metabolites and for neutralizing and eliminating toxic substances. This elimination occurs in the form of bile, an exocrine secretion of the liver which is important in lipid digestion. The basic structural component of the liver is the liver cell or hepatocyte. In light microscope, structural units called classic liver lobules can be seen. The liver lobule is formed of a polygonal mass of tissue about 0.7 × 2 mm in size (Figure 4).

**Figure 5:**
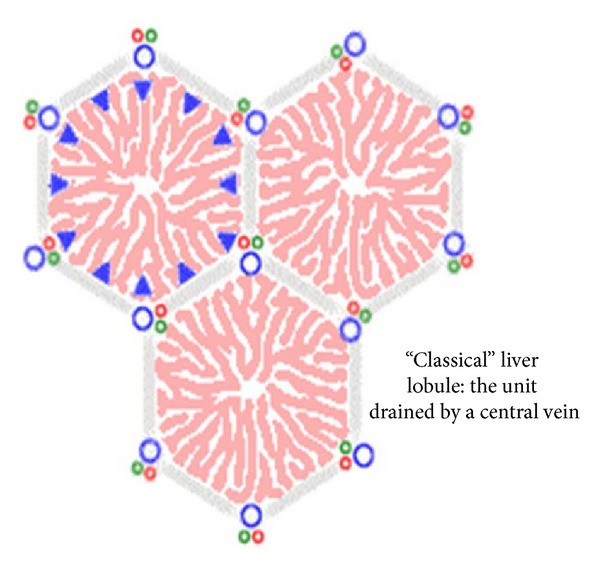


In certain animals (e.g., the pig), lobules are separated from each other by a layer of connective tissue. In humans, it is difficult to establish the exact limits between different lobules since they are in close contact in most of their extent (Figure 5). In some regions, the lobules are demarcated by connective tissue containing bile ducts, lymphatic vessels, nerves, and blood vessels. These regions, located at the corners of the lobules and occupied by portal triads, are called portal spaces. The human liver contains 3–6 portal triads per lobule, each with a venule (a branch of the portal vein); an arteriole (a branch of the hepatic artery); a duct (part of the bile duct system); and lymphatic vessels. The venule is usually the largest of these structures, containing blood from the superior and inferior mesenteric and splenic veins. An arteriole contains blood from the celiac trunk of the abdominal aorta.

The hepatocytes in the liver lobule are radially disposed and arranged like the bricks of a wall. These cellular plates are directed from the periphery of the lobule to its center and anastomose freely, forming a labyrinthine and sponge-like structure. The space between these plates contains capillaries, the liver sinusoids [2]. Portal and arterial blood mixes in the sinusoids and flows past hepatocytes, draining through a central vein from each lobule that leads ultimately to the hepatic veins. Bile from the lobules drains into the interlobular branches of the bile duct by way of bile canaliculi. The hepatic lobules act as endocrine and exocrine glands. In endocrine secretion, hepatocytes take up and secrete molecules into the sinusoids [1].

The liver has an extraordinary capacity for regeneration. Hence, the loss of hepatic tissue by surgical removal or from the action of toxic substances is restored. The liver performs its metabolic functions with the aid of various enzymes fixed inside liver cells. These liver cells line many capillaries (hepatic sinusoids) through which the total hepatic blood flow is manifolded, whereby exchange of substances between blood flow and cells is facilitated. The interplay of the unidirectional blood flow with local metabolism generates concentration gradients of blood-borne substances (such as oxygen) between the inlet and the outlet of the liver. The unidirectionality of that blood, that is, the blood flows form the portal triads to the central vein (Figure 5), has a major influence on the mathematical structure of the model, which appears to be capable of describing the formation of zones with a jump discontinuity at a certain distance along a capillary [3].

Several metabolic functions of the liver have been found to be organized in spatial zones arranged in relation to the direction of hepatic blood flow, in such a way that some enzymes act almost wholly upstream others [3]. Bass et al. [3] attributed such distributions of enzymes activities to distributions of cell types. For the simplest case of two enzymes, there are two corresponding cell types, each containing only one of the enzymes; separate metabolic zones occur when all cells of one type are located upstream all cells of the other type. Furthermore, it was reported in [3] that each cell type reproduces itself by division. The mathematical model was discussed in [3], but for convenience the main steps in its derivation are repeated in the next section. The mathematical model describing the formation of liver zones is a system of nonlinear integropartial differential equations. The objective of this paper is to apply Adomian's decomposition method to the system in order to find its stationary solutions (as the time tends to infinity) and the general solutions (at any position *x* and any time *t*) for arbitrary initial conditions.

## 2. Mathematical Modelling and Solutions

### 2.1. Mathematical Formulation

About 1100 milliliters of blood flows from the portal vein into the liver sinusoids (Figure 6) each minute, and approximately an additional 350 milliliters flows into the sinusoids from the hepatic artery, the total averaging is about 1450 mL/min. This amounts to about 29% of the resting cardiac output. As the many capillaries comprising the liver are similar and act essentially in parallel, Bass et al. [3] modelled a representative capillary lined with cells of two kinds. It was suggested to put the *x*-axis along the blood flow, with inlet at *x* = 0 and outlet at *x* = *L* [3]. The density of cells of the first kind is defined by *ρ*_1_(*x*, *t*) as a continuous representation of the number of cells of the first kind per unit length of capillary at time *t* at the position *x*. The density *ρ*_2_(*x*, *t*) of cells of the second kind is defined analogously. The total cell density *ρ*_1_ + *ρ*_2_ cannot exceed some fixed maximum density *σ* of cell sites, as division of the cell is limited by the familiar phenomenon of contact inhibition. The local rate of change ∂*ρ*_1_/∂*t* of the density of cells of the first kind is assumed to consist of a growth rate term proportional to *ρ*_1_ (self-generation) and to the density of sites available, *σ* − *ρ*_1_ − *ρ*_2_, and of a death rate term proportional to *ρ*_1_, with a coefficient *β*_1_(*c*) > 0 dependent on the local concentration *c* of a controlling blood-borne substance. In what follows, for definiteness, oxygen is taken as that substance. Then

(1) $$\begin{array}{c} \frac{\partial \rho_{1}}{\partial t}=K_{1}\rho_{1}\left(\sigma -\rho_{1}-\rho_{2}\right)-\beta_{1}\left(c\right)\rho_{1}, \end{array}$$

with a constant coefficient *K*_1_ > 0. A similar equation for *ρ*_2_ is obtained from (1) by interchanging the suffices 1, 2. So

(2) $$\begin{array}{c} \frac{\partial \rho_{2}}{\partial t}=K_{2}\rho_{2}\left(\sigma -\rho_{1}-\rho_{2}\right)-\beta_{2}\left(c\right)\rho_{2}. \end{array}$$

Let *f* be the steady rate of blood flow through the capillary. If oxygen is transported in the *x*-direction predominantly by convection with the blood and used up by the two cell types at the rates *k*_1_ *ρ*_1_ and *k*_2_ *ρ*_2_ (with positive constants *k*_1_, *k*_2_), then changes in *c* caused by changes in *ρ*_1_ and *ρ*_2_ are quasisteady. Therefore, *c* satisfies

(3) $$\begin{array}{c} f\frac{\partial c}{\partial x}=-k_{1}\rho_{1}-k_{2}\rho_{2}. \end{array}$$

If (3) is integrated, then

(4) $$\begin{array}{c} c\left(x,t\right)=c_{0}-\frac{1}{f}\int_{0}^{x}\left[k_{1}\rho_{1}\left(\xi ,t\right)+k_{2}\rho_{2}\left(\xi ,t\right)\right]d\xi , \end{array}$$

where *c*_0_ is the steady oxygen concentration in the blood entering the liver. It is assumed that as oxygen concentration falls, the death rate of cells increases (*dβ*_1_(*c*)/*dc* ≤ 0, *dβ*_2_(*c*)/*dc* ≤ 0), though not necessarily equally for both cell types. It is assumed that *β*_1_(*c*) has the following form (similarly for *β*_2_(*c*)):

(5) $$\begin{array}{c} \beta_{1}\left(c\right)=\mu_{1}+\nu_{1}\left(c_{0}-c\right), \end{array}$$

where

(6) $$\begin{array}{c} \mu_{1}=\beta_{1}\left(c_{0}\right)\geq 0,\;\;\nu_{1}=-{\frac{d\beta_{1}}{dc}|}_{c_{0}}\geq 0. \end{array}$$

Introducing (4) and (5) into (1) and (2), we arrive at the pair of equations

(7) ∂ρ1∂t=ρ1[K1(σ−ρ1−ρ2)−μ1−ν1f∫0x(k1ρ1+k2ρ2)dξ],∂ρ2∂t=ρ2[K2(σ−ρ1−ρ2)−μ2−ν2f∫0x(k1ρ1+k2ρ2)dξ].

If *K*_1_ *σ* ≤ *μ*_1_, then *ρ*_1_(*x*, *t*) → 0 as *t* → *∞* for all *x*, and similarly for *ρ*_2_. Therefore, it is assumed that *K*_1_ *σ* > *μ*_1_ and *K*_2_ *σ* > *μ*_2_. For similar reasons, it is assumed that at least one of *ν*_1_ and *ν*_2_ is positive (say *ν*_1_). It is noted at once that unless the first cell type is inevitably to die out, its greatest possible specific growth rate *K*_1_ *σ* must exceed its least possible specific death rate *μ*_1_. Similar remarks apply for the second cell type, and accordingly it is assumed in [3] that

(8) $$\begin{array}{c} K_{1}\sigma >\mu_{1},\;\;K_{2}\sigma >\mu_{2}. \end{array}$$

To obtain some preliminary heuristic ideas about the formation of zones in their model, Bass et al. [3] supposed that (7) admits solutions which at all finite times are everywhere positive and satisfy (*ρ*_1_ + *ρ*_2_) < *σ*. For such solutions, (7) can be written in the form

(9) ∂∂tln⁡ρ1=K1(σ−ρ1−ρ2)−μ1−ν1f∫0x(k1ρ1+k2ρ2)dξ,∂∂tln⁡ρ2=K2(σ−ρ1−ρ2)−μ2−ν2f∫0x(k1ρ1+k2ρ2)dξ.

Multiplying the first equation in (9) by *K*_2_ and the second by *K*_1_, we have

(10) ∂∂tln⁡ρ1K2=K1K2(σ−ρ1−ρ2)−μ1K1−ν1K1f∫0x(k1ρ1+k2ρ2)dξ,∂∂tln⁡ρ2K1=K1K2(σ−ρ1−ρ2)−μ2K2−ν2K2f∫0x(k1ρ1+k2ρ2)dξ.

### 2.2. The Stationary Solutions

For such solutions, we can combine (10) in the form

(11) $$\begin{array}{c} \frac{\partial }{\partial t}\left[\ln\left(\frac{\rho_{1}^{K_{2}}}{\rho_{2}^{K_{1}}}\right)\right]=A-\frac{B}{f}\int_{0}^{x}\left(k_{1}\rho_{1}+k_{2}\rho_{2}\right)d\xi , \end{array}$$

where

(12) $$\begin{array}{c} A=\mu_{2}K_{1}-\mu_{1}K_{2},\;\;B=\nu_{1}K_{2}-\nu_{2}K_{1}. \end{array}$$

Suppose that *μ*_2_ *K*_1_ > *μ*_1_ *K*_2_, *ν*_1_ *K*_2_ > *ν*_2_ *K*_1_, or

(13) $$\begin{array}{c} \frac{\mu_{2}}{K_{2}}>\frac{\mu_{1}}{K_{1}},\;\;\frac{\nu_{2}}{K_{2}}<\frac{\nu_{1}}{K_{1}}, \end{array}$$

so that the constants *A* and *B* are positive. Since the integral in (11) is bounded above by (*k*_1_ + *k*_2_)*xσ*, the right-hand side of (11) is positive at all times for sufficiently small *x*, where

(14) $$\begin{array}{c} x<\frac{Af}{\left(k_{1}+k_{2}\right)\sigma B}. \end{array}$$

Volterra's argument [3] then applies: as *t* → *∞*, *ρ*_1_^*K*_2_^/*ρ*_2_^*K*_1_^ → *∞*, and with *ρ*_1_ bounded above by *σ*, *ρ*_2_ must tends to zero. It is then plausible that for these values of *x* in (14), *ρ*_1_ will approach a stationary form determined from the first equations of (7) with *ρ*_2_ = 0, namely [3, 4],

(15) $$\begin{array}{c} K_{1}\left(\sigma -\rho_{1}\right)-\mu_{1}-\frac{\nu_{1}}{f}\int_{0}^{x}k_{1}\rho_{1}d\xi =0. \end{array}$$

In order to solve this equation by Adomian's decomposition method [5–13], we put the equation in the form

(16) $$\begin{array}{c} \rho_{1}=\frac{c_{1}}{K_{1}}-\left(\frac{\nu_{1}k_{1}}{fK_{1}}\right)\int_{0}^{x}\rho_{1}d\xi , \end{array}$$

where

(17) $$\begin{array}{c} c_{i}=K_{i}\sigma -\mu_{1},\;i=1,2. \end{array}$$

According to Adomian's method, *ρ*_1_ is assumed as

(18) $$\begin{array}{c} \rho_{1}=\sum_{n=0}^{\infty }\rho_{1n}. \end{array}$$

Substituting (18) into (16), we obtain

(19) $$\begin{array}{c} \sum_{n=0}^{\infty }\rho_{1n}=\frac{c_{1}}{K_{1}}-\left(\frac{\nu_{1}k_{1}}{fK_{1}}\right)\sum_{n=0}^{\infty }\int_{0}^{x}\rho_{1n}d\xi . \end{array}$$

Let

(20) $$\begin{array}{c} \rho_{10}=\frac{c_{1}}{K_{1}}. \end{array}$$

Then the solution can be elegantly computed by using the recurrence relation

(21) $$\begin{array}{c} \rho_{1(n+1)\;}=-\frac{\nu_{1}k_{1}}{fK_{1}}\int_{0}^{x}\rho_{1n}d\xi ,\;n\geq 0. \end{array}$$

This gives

(22) ρ11(x)=(−ν1k1fK1)c1K1x,ρ12(x)=(−ν1k1fK1)2c1K1x22!,ρ13(x)=(−ν1k1fK1)3c1K1x33!,⋮ρ1n(x)=(−ν1k1fK1)nc1K1xnn!, n≥0.

According to (18), we obtain *ρ*_1_ in the form

(23) $$\begin{array}{c} \rho_{1}\left(x\right)=\rho_{1}^{*}\left(x\right)=\frac{c_{1}}{K_{1}}\exp\left[-\frac{\nu_{1}k_{1}}{fK_{1}}x\right]. \end{array}$$

Set *ρ*_2_ equal to zero and *ρ*_1_ equal to *ρ*_1_*, then (11) becomes

(24) $$\begin{array}{c} \frac{\partial }{\partial t}\left[\ln\left(\frac{\rho_{1}^{K_{2}}}{\rho_{2}^{K_{1}}}\right)\right]=A-\frac{Bc_{1}}{\nu_{1}}\left[1-\exp\left(-\frac{\nu_{1}k_{1}}{fK_{1}}x\right)\right]. \end{array}$$

We note that the right-hand side of (24) decreases with increasing *x* and reaches zero at a value *x* = x* determined by

(25) $$\begin{array}{c} \exp\left(\frac{\nu_{1}k_{1}x^{*}}{fK_{1}}\right)=\frac{Bc_{1}}{K_{1}\left(\nu_{1}c_{2}-\nu_{2}c_{1}\right)}. \end{array}$$

Provided that

(26) $$\begin{array}{c} \nu_{1}c_{2}>\nu_{2}c_{1}. \end{array}$$

The point *x* = x* determined by (25) lies in the interval (0, *L*) of interest provided that

(27) $$\begin{array}{c} \exp\left(\frac{\nu_{1}k_{1}L}{fK_{1}}\right)>\frac{Bc_{1}}{K_{1}\left(\nu_{1}c_{2}-\nu_{2}c_{1}\right)}. \end{array}$$

Under these conditions, it is then reasonable to suppose that, for *x* > x*, the right-hand side of (11) will in fact be negative for sufficiently large values of *t* [3]. Volterra's argument then indicates that we can expect to find *ρ*_1_ → 0 as *t* → *∞* for *x* > x*. Furthermore, we may also expect that, for *x* > x*, *ρ*_2_ will approach a stationary form determined from the second equation of (7) by

(28) $$\begin{array}{c} K_{2}\left(\sigma -\rho_{2}\right)-\mu_{2}-\frac{\nu_{2}}{f}\left[\int_{0}^{x^{*}}k_{1}\rho_{1}^{*}d\xi +\int_{x^{*}}^{x}k_{2}\rho_{2}d\xi \right]=0. \end{array}$$

This equation can be solved by Adomian's method; we rewrite the equation in the form

(29) $$\begin{array}{c} \rho_{2}+\frac{\nu_{2}k_{2}}{fK_{2}}\int_{x^{*}}^{x}\rho_{2}d\xi =D, \end{array}$$

where

(30) $$\begin{array}{c} D=\frac{\nu_{1}c_{2}-\nu_{2}c_{1}}{B}. \end{array}$$

We assume that

(31) $$\begin{array}{c} \rho_{2}=\sum_{n=0}^{\infty }\rho_{2n}. \end{array}$$

Let *ρ*_20_ = *D*, then the solution can be computed by using the recurrence relation

(32) $$\begin{array}{c} \rho_{2(n+1)}=-\frac{\nu_{2}k_{2}}{fK_{2}}\int_{x^{*}}^{x}\rho_{2n}d\xi ,\;n\geq 0. \end{array}$$

This gives

(33) ρ21=−−ν2k2fK2D(x−x∗),ρ22=(−ν2k2fK2)2D(x−x∗)22!,⋮ρ2n=(−ν2k2fK2)nD(x−x∗)nn!, n≥0.

Therefore

(34) $$\begin{array}{c} \rho_{2}=\rho_{2}^{*}\left(x\right)=D\exp\left[-\frac{\nu_{2}k_{2}}{fK_{2}}\left(x-x^{*}\right)\right],\;x>x^{*}. \end{array}$$

So, as *t* → *∞*, the formation of liver zones can be described as follows:

(35) $$\begin{array}{c} \rho_{1}=\rho_{1}^{*}\left(x\right),\;\rho_{2}=0,\;0\leq x<x^{*},\\ \rho_{1}=0,\;\rho_{2}=\rho_{2}^{*}\left(x\right),\;x^{*}<x\leq L. \end{array}$$

## 3. Analytical Solutions

 In applied mathematics, Adomian's decomposition method is an effective procedure to obtain analytic and approximate solutions for different types of equations. This method is used here to obtain a general solution for the system (7). Following Bass et al. [3], we define new variables

(36) $$\begin{array}{c} t^{\prime }=c_{1}t,\;\;x^{\prime }=\frac{\nu_{1}k_{1}}{fK_{1}}x,\;\;v_{i}\left(t^{\prime },x^{\prime }\right)=\frac{K_{1}}{c_{1}}\rho_{i}\left(t,x\right) \end{array}$$

and new parameters

(37) $$\begin{array}{c} \theta =\frac{k_{2}}{k_{1}},\;\;\gamma =\frac{K_{2}}{K_{1}},\;\;\lambda =\frac{K_{1}c_{2}}{K_{2}c_{1}},\;\;\eta =\frac{\nu_{2}K_{1}}{\nu_{1}K_{2}}. \end{array}$$

Then (7) becomes, on dropping at once the primes from the new independent variables,

(38) ∂v1∂t=v1[1−v1−v2−∫0x[v1(t,ξ)+θv2(t,ξ)]dξ],∂v2∂t=γv2[λ−v1−v2−η∫0x[v1(t,ξ)+θv2(t,ξ)]dξ],

with constant parameters

(39) $$\begin{array}{c} \theta >0,\;\;\gamma >0,\;\;\lambda >0,\;\;\eta \geq 0. \end{array}$$

The spatial interval of interest is now [0, Λ], where Λ = (*ν*_1_ *k*_1_/*fK*_1_)*L*, and then we have [3]

(40) $$\begin{array}{c} \eta <\lambda <1,\;\;\ln\left(\frac{1-\eta }{\lambda -\eta }\right)<\Lambda . \end{array}$$

The stationary solutions become

(41) $$\begin{array}{c} v_{1}=e^{-x},\;v_{2}=0,\;0\leq x<x^{*},\\ v_{1}=0,\;v_{2}=\left(\frac{\lambda -\eta }{1-\eta }\right)e^{-\eta \theta (x-x^{*})},\;x^{*}<x\leq \Lambda , \end{array}$$

where now

(42) $$\begin{array}{c} x^{*}=\ln\left(\frac{1-\eta }{\lambda -\eta }\right). \end{array}$$

For searching analytical solutions, we firstly rewrite the system we want to solve as two separate integro-partial differential equations:

(43) $$\begin{array}{c} \frac{\partial v_{1}}{\partial t}=v_{1}\left[1-v_{1}-v_{2}-\int_{0}^{x}\left(v_{1}+\theta v_{2}\right)d\xi \right], \end{array}$$

(44) $$\begin{array}{c} \frac{\partial v_{2}}{\partial t}=\gamma v_{2}\left[\lambda -v_{1}-v_{2}-\eta \int_{0}^{x}\left(v_{1}+\theta v_{2}\right)d\xi \right]. \end{array}$$

According to the decomposition method, we assume that

(45) $$\begin{array}{c} v_{1}=\sum_{n=0}^{\infty }v_{1n},\;\;v_{2}=\sum_{n=0}^{\infty }v_{2n}. \end{array}$$

Equation (43) can be put in the following operator form:

(46) Ltv1=v1−v12−v1v2−v1∫0x(v1(t,ξ)+θv2(t,ξ))dξ,                      Lt=∂∂t.

Applying the inverse operator *L*_*t*_^−1^[·] = ∫_0_^*t*^[·]*dt*, on both sides of this equation, yields

(47) v1−v1(x,0)=Lt−1v1−Lt−1v12−Lt−1v1v2−Lt−1v1∫0x(v1+θv2)dξ.

Substituting (45) into (47), we obtain

(48) v1=v1(x,0)+Lt−1∑n=0∞v1n−Lt−1∑n=0∞∑k=0nv1kv1(n−k)−Lt−1∑n=0∞∑k=0nv1kv2(n−k)−Lt−1∑n=0∞v1n∫0x∑n=0∞(v1n+θv2n)dξ.

Now, the solution *v*_1_ can be evaluated through the recursive scheme:

(49) v10(x,t)=v1(x,0),v1(n+1)(x,t)=Lt−1v1n−Lt−1(∑k=0nv1kv1(n−k))−Lt−1(∑k=0nv1kv2(n−k))−Lt−1∑k=0nv1k×∫0x(v1(n−k)+θv2(n−k))dξ, n≥0.

By similar analysis, we can get the solution *v*_2_ by the recursive scheme:

(50) v20(x,t)=v2(x,0),v2(n+1)(x,t)=γλLt−1v2n−γLt−1(∑k=0nv1kv2(n−k))−γLt−1(∑k=0nv2kv2(n−k))−γλLt−1∑k=0nv2k×∫0x(v1(n−k)+θv2(n−k))dξ, n≥0.

For simplicity, we assume that the two types of the liver cells have the same distribution along the hepatic capillary at *t* = 0; that is,

(51) $$\begin{array}{c} v_{1}\left(x,0\right)=v_{2}\left(x,0\right);\\ \text{that}\;\text{is},v_{10}\left(x\right)=v_{20}\left(x\right)=v_{0}\left(x\right)\left(\text{say}\right). \end{array}$$

By this, we can get the first few terms of Adomian's series from the recurrence relations (49) and (50) as follows:

(52) v11(x,t)=v0[1−2v0−(1+θ)I1(x)]t,v21(x,t)=γv0(λ−2v0−η(1+θ)I1(x))t,v12=v0{[1−3v0−(1+θ)I1(x)][1−2v0−(1+θ)I1(x)]  −γv0[λ−2v0−η(1+θ)I1(x)]−(1+γθλ)I1(x)  +2(1+γθ)I2(x)(1+γηθ)(1+θ)I3(x)}t22!,v22={[γλ−3γv0−γη(1+θ)I1(x)] ×[γλv0−2γv02−γη(1+θ)v0I1(x)] −[γv02−2γv03−γ(1+θ)v02I1(x)] −γη(1+γθλ)v0I1(x)+2γη(1+γθ)v0I2(x) +γη(1+θ)(1+γηθ)v0I3(x)}t22!,

where

(53) $$\begin{array}{c} I_{1}\left(x\right)=\int_{0}^{x}v_{0}d\xi ,\;\;I_{2}\left(x\right)=\int_{0}^{x}v_{0}^{2}d\xi ,\\ I_{3}\left(x\right)=\int_{0}^{x}I_{1}\left(\xi \right)v_{0}d\xi . \end{array}$$

## 4. Remarks

Here, we indicate that at particular values of the parameters *γ*, *λ*, and *η*, the solutions *v*_1_ and *v*_2_ are equivalent. In order to do this, we prefer to put the solutions *v*_1_ and *v*_2_ in the form

(54) v1(x,t)=α0(x)+α1(x)t+α2(x)t2+⋯,v2(x,t)=β0(x)+β1(x)t+β2(x)t2+⋯,

where

(55) α0(x)=β0(x)=v0(x),α1(x)=v0[1−2v0−(1+θ)I1(x)],α2(x)=12!v0[1−3v0−(1+θ)I1(x)]×[1−2v0−(1+θ)I1(x)]−γv0[λ−2v0−η(1+θ)I1(x)]−(1+γθλ)I1(x)+2(1+γθ)I2(x)+(1+γηθ)(1+θ)I3(x),β1(x)=γv0[λ−2v0−(1+θ)I1(x)],β2(x)=12!{[γλ−3γv0−γη(1+θ)I1(x)]  ×[γλv0−2γv02−γη(1+θ)v0I1(x)]  −[γv02−2γv03−γ(1+θ)v02I1(x)]  −η(1+γθλ)v0I1(x)+2γη(1+γθ)v0I2(x)  +γη(1+θ)(1+γηθ)v0I3(x)}.

Firstly, substituting *γ* = *λ* = *η* = 1 into the original equations (38), we obtain

(56) 1v1∂v1∂t=1−v1−v2−∫0x(v1+θv2)dξ,1v2∂v2∂t=1−v1−v2−∫0x(v1+θv2)dξ.

We can combine these equations in the form

(57) $$\begin{array}{c} \frac{\partial }{\partial t}\left[\ln v_{1}\left(x,t\right)\right]=\frac{\partial }{\partial t}\left[\ln v_{2}\left(x,t\right)\right]. \end{array}$$

By integrating both sides with respect to *t* from 0 to *t*, we get

(58) $$\begin{array}{c} \ln\left[\frac{v_{1}\left(x,t\right)}{v_{0}\left(x\right)}\right]=\ln\left[\frac{v_{2}\left(x,t\right)}{v_{0}\left(x\right)}\right], \end{array}$$

where we used the relation *v*_1_(*x*, 0) = *v*_2_(*x*, 0) = *v*_0_(*x*). Thus, *v*_1_(*x*, *t*) = *v*_2_(*x*, *t*). Now, substituting *γ* = *λ* = *η* = 1 into (55), we can easily observe that

(59) $$\begin{array}{c} \alpha_{0}\left(x\right)=\beta_{0}\left(x\right),\;\;\alpha_{1}\left(x\right)=\beta_{1}\left(x\right),\;\;\alpha_{2}\left(x\right)=\beta_{2}\left(x\right), \end{array}$$

which leads also to *v*_1_(*x*, *t*) = *v*_2_(*x*, *t*).

## 5. Conclusion

 In this paper, the Adomian decomposition method has been applied successfully to a system of nonlinear integro-partial differential equations describing the formation of liver zones. As time tends to infinity, the stationary solutions are obtained in exact forms by using Adomian's method, where full agreement with those obtained in the literature has been achieved. Also, at any time of the liver regeneration process, the analytical solutions are obtained explicitly in series form. Finally, the current solutions may shed some light on the mathematical aspects of the formation of liver zones and also on describing the distribution of the two types of the liver cells.

## Figures and Tables

**Figure 6:**